# Effect of Low-Magnitude Whole-Body Vibration Combined with Alendronate in Ovariectomized Rats: A Random Controlled Osteoporosis Prevention Study

**DOI:** 10.1371/journal.pone.0096181

**Published:** 2014-05-05

**Authors:** Guo-Xian Chen, Shuai Zheng, Shuai Qin, Zhao-Ming Zhong, Xiu-Hua Wu, Zhi-Ping Huang, Wei Li, Ruo-Ting Ding, Hui Yu, Jian-Ting Chen

**Affiliations:** 1 Department of Orthopedic Spinal Surgery, Nanfang Hospital, Southern Medical University, Guangdong Province, Guangzhou, China; 2 Department of Orthopedic, the First Hospital of Putian City, Fujian Province, Putian City, China; 3 Department of ophthalmology, The People's Hospital of Zhuhai, Zhuhai, China; INSERM U1059/LBTO, Université Jean Monnet, France

## Abstract

**Background:**

Alendronate (ALE) is a conventional drug used to treat osteoporosis. Low-magnitude whole-body vibration (WBV) exercise has been developed as a potential treatment for osteoporosis. The aim of this study was to investigate whether low-magnitude WBV could enhance the protective effect of ALE on bone properties in ovariectomized rats.

**Methods:**

A total of 128 Sprague-Dawley rats were randomly divided into five groups (SHAM, OVX+VEH, OVX+WBV, OVX + ALE, OVX+WBV+ALE). The level of WBV applied was 0.3 g at 45–55 Hz for 20 min/day, 5 day/week and for 3 months. ALE was administered in dose of 1 mg/Kg once a week. Every four weeks eight rats from each group were sacrificed and their blood and both tibiae were harvested. The expression of osteocalcin and CTX in serum was measured by enzyme-linked immunosorbent assay (ELISA) and the tibiae were subjected to metaphyseal three-point bending and μCT analysis.

**Results:**

Osteocalcin rose after ovariectomy and was not appreciably changed by either alendronate or WBV alone or in combination. Alendronate treatment significantly prevented an increase in CTX. WBV alone treatment did not alter this effect. Compared with the OVX+WBV group, nearly all tested indices such as the BV/TV, TV apparent, Tb.N, Tb.Th, and Conn.D were higher in the OVX+ALE group at week 12.Compared with the OVX+WBV group, certain tested indices such as BV/TV, TV apparent, Tb.N, and Con.D, were higher in the OVX+WBV+ALE group at week 12. At week 12, tibiae treated with WBV+ALE exhibited a significantly higher F_max_ compared to the OVX+VEH group, and a significant difference was also found in energy absorption between the OVX+WBV+ALE and OVX+VEH groups.

**Conclusions:**

Compared with the WBV, ALE was more effective at preventing bone loss and improved the trabecular architecture. However, WBV enhanced the effect of alendronate in ovariectomized rats by inducing further improvements in trabecular architecture.

## Introduction

With an increasing aging population worldwide, osteoporosis has become a growing public concern [1]. Osteoporosis is characterized by an imbalance between bone formation and absorption. Osteoporosis causes not only osteoporotic fracture and postural deformities, but also causes chronic and acute back pain, which can potentially results in disability and deterioration in the quality of life (QOL) in elderly females [2]–[3]. In humans, menopause is accompanied by increased bone turnover, which can result in bone loss of approximately 2%/year over five years and an increased susceptibility to fractures [4]–[5]. Moreover, osteoporotic fractures can increase the financial burden of medical care [6]–[7]. Thus, there is an urgent need to develop effective and safe approaches to increase bone strength.

Bisphosphonates, such as alendronate (ALE), are potent inhibitors of osteoclast-mediated bone resorption that suppress bone turnover through their ability to prevent the initiation of new erosion sites and to reduce ongoing excavation [8]–[9]. ALE is therapeutic candidate for preventing bone loss in estrogen-deficient states [10]–[11]. ALE has been proven effective in rats in preventing bone loss associated with immobilization by reducing bone resorption [12]. Previous evidence suggests that ALE can reduce back pain and increase activities of daily living in elderly females [3]. Furthermore, it is well-known that many drugs, including ALE, have corresponding adverse reactions, which limit certain patients from taking this drug long-term.

Recently, low-magnitude whole-body vibration (WBV) exercise has been developed as a new and effective method for treating osteoporosis [13]. Many studies have shown that mechanical stimulation via WBV is beneficial for maintaining and/or enhancing bone mass [14]–[16], and improving neuromuscular function [17]–[18], as well as other physiological benefits. The attractiveness of such a therapy relies on its ability to be applied in a low-impact manner, which is critical for individuals with impaired mobility and attenuated muscle strength [19]–[20]; for example elderly or diseased people.

However, to the best of our knowledge, there are no published studies investigating the effect of WBV combined with ALE in ovariectomized rats. The aim of this study was to investigate the effect of WBV combined with ALE in ovariectomized rats and to determine whether WBV exercise would enhance the effect of ALE on bone turnover and bone properties in ovariectomized rats.

## Materials and Methods

### Ethics statement

This study was performed in strict accordance with the guidelines of caring for laboratory animals of the Ministry of Science and Technology of the People's Republic of China. All animal procedures were approved by the Committee on the Ethics of Animal Experiments of Southern Medical University. All surgeries were performed using isoflurane anesthesia, and every effort were made to minimize suffering. At the end of the experiment isoflurane was used for animal euthanasia.

### Specimens

A total of 128 (32 in the Sham group, and 24 in each treatment groups) female Sprague-Dawley rats (eight weeks old, purchased from the Laboratory Animals center of Southern Medical University, acclimate to one week in our facility) were used in this experiment. The rats were raised in a 12-h light/dark cycle and fed a standard rodent diet (provided by Laboratory Animals center of Southern Medical University) and water ad libitum. The experimental animals were randomly assigned to five groups (Sham, OVX+VEH, OVX+WBV, OVX+ALE, OVX+WBV+ALE) according to body weight with either skin incision and suture, ovariectomized with VEH (Vehicle, in this study it was PBS which was used to make RSA solution.), ovariectomized with WBV (0.3 g at 45–55 Hz for continuous 20 min/day, 5 day/week, for 3 months), ovariectomized with ALE (intragastric administration 1 mg/Kg, once a week) or ovariectomized with WBV and ALE.

During the experimental period, the body weight of the rats in all groups was measured using electronic scale every four weeks during the experimental period. The rats were sacrificed at four predetermined time points for the Sham group (0, 4, 8 and 12 weeks) and three time points for the remaining four groups (4, 8 and 12 weeks). There were 32 animals in the Sham groups and 24 animals in each treatment groups. At sacrifice, whole blood was collected with a cardiac puncture to determine the serum osteocalcin (OC) and CTX (C-terminal cross-linked telopeptides of type I collagen, CTX) using standard laboratory techniques. The bilateral tibiae were collected, with the soft tissues being thoroughly removed and wrapped in normal saline-soaked gauze and stored at −20°C until further use for biomechanical testing and micro-CT scanning.

### Ovariectomy procedure

In this study, surgery was performed on 96 Sprague-Dawley rats to remove the ovaries. Sham surgeries (where the surgery was performed, but the ovaries were not removed) were performed on 36 rats (Sham group). The animals were anesthetized using isoflurane. The lower abdomen was surgically scrubbed and an incision was made to expose the ovaries and uterus. The ovaries were removed, and subsequently, the muscles and skin were sutured. All animals recovered from the surgery without incident. Uterine weight was measured using electronic scale.

### Serum markers

Blood was obtained from the rat cardiac puncture prior to their sacrifice, and the specimens were centrifuged to isolate the serum and stored at −80°C, until the samples were further analyzed for osteocalcin (OC bone formation markers) and CTX, bone resorption markers according to the manufacturer's instructions. The two markers were measured using an enzyme-linked immunosorbent assay (ELISA Cusabio, Wu Han, China). The absorbance at 450 nm was measured using a spectrophotometric plate reader.

### Micro-CT scanning

The left tibiae were stored in the saline soaked gauze at −20°C. The micro-architecture of the trabecular bone were assessed by using a high-resolution micro-CT system (μCT80, Scanco Medical AG, Bassersdorf, Switzerland) equipped with a 12 µm focal spot microfocus X-ray tube as the source.

**Figure 1:**
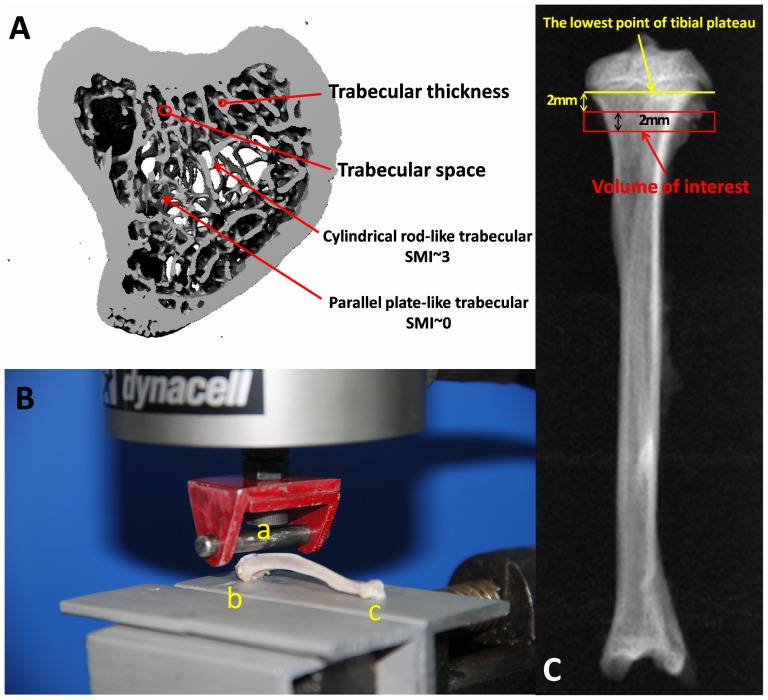
General introduction of the experimental methods of μCT and the three-point bending test. (A) Experimental parameters of the rat tibia scanned using μCT. (B) Details on the experimental design of the three-point bending test consisting of an aluminum block and rounded edge-free notches. (C) The volume of test samples using μCT and the three-point bending test.

The bone morphology and density parameters were measured at one location in the left tibia. The scanning region used for the structural evaluation was defined as a 2-mm region, which started from 2 mm distal to the proximal growth plate (Fig. 1 A and C). The tissue volume (TV) was determined by thresholding the image at 0.50 g/cm^3^, and quantifying the enclosed voxels with an additional dilation-erosion step to fill in the occasional small voids in the cortical wall. The bone volume (BV) was a subset of the tissue volume threshold. The ratio of BV/TV represented the normalized bone volume. Low-density foam was used to position the specimen tightly in the sample holder to ensure that there would be no relative movement between the specimen and the sample holder during the scan. The resulting grayscale images obtained had an isotropic voxel size of 12 µm, and the X-ray tube was operated at 55 k V p and 49 µA.

### Biomechanical testing

The mechanical properties of the tibiae were investigated using the three-point bending method using a miniature Instron materials testing machine (Electroplus E1000 Test System) with a 2000 N load cell (Fig. 1B). The tibiae were thawed at room temperature for 30 min prior to testing the region of interest, as shown in Fig. 1C. The samples, which were continuously moistened with isotonic saline solution during the test, were placed on a base consisting of an aluminum block with one-rounded edge-free notch on the top (Fig. 1B). The end of the dorsal proximal tibia (the two condyles) was placed in one of the notches (b in Fig. 1B). Because of its curved shape, the posterior distal diaphysis was rested on the other side of base (c in Fig. 1B). During the breaking test, the proximal tibia could not slip due to the notch on the base, but it was able to lengthen along the diaphyseal axis. The tip of the stamp consisted of an axle-led aluminum roller (8 mm high, 8 mm wide, 8 mm in diameter). A 2-mm-wide and 1-mm-deep circular notch with rounded edges was located in the center of the roller (a in Fig. 1B). The speed of the feed motion was 5 mm/min with 5% strain rate, and the test was automatically stopped after a loss of strength at >20 N or a linear change of >2 mm to avoid shattering the tibial specimens. The maximum load (Fmax), energy absorption and stiffness (S) were collected using Bluehill 2 (Version 2.28.832, Instron, a Division of Illinois Tool Works Inc). The experiment was blinded with regard to the association between the bones and animal groups.

### Statistical analyses

The results were expressed as the means ± standard deviation (SD). Significant differences between the means for different groups were compared using one-way analysis of variance (ANOVA). Multiple comparisons were performed using the LSD method or Dunnett's C method. Comparisons of several treatments with the control group were evaluated using the Dunnett's T3 procedure. All statistical analyses were performed using SPSS 13.0 software. A significance level of P<0.05 was used for all comparisons.

## Results

### Monitoring

**Figure 2:**
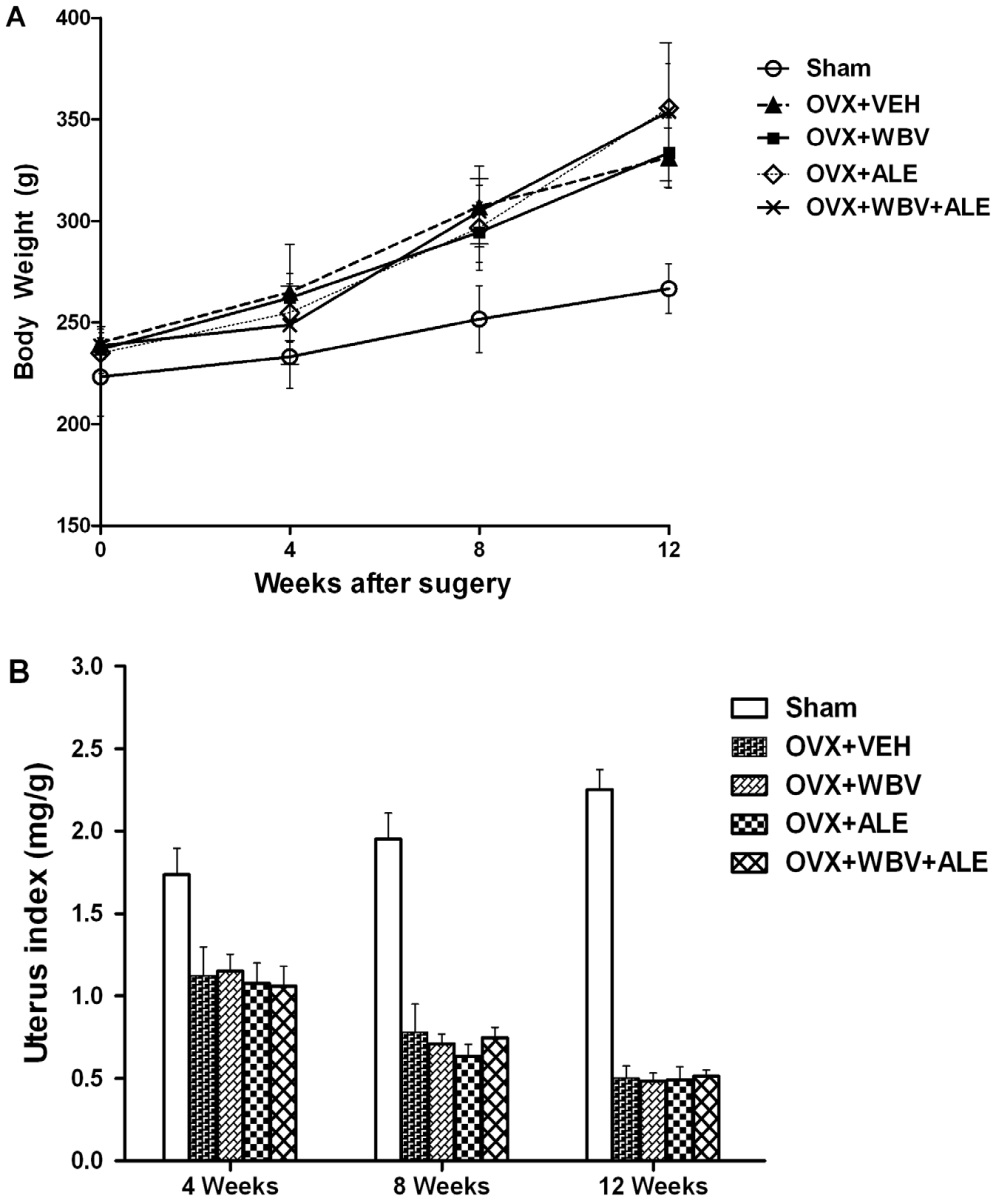
The body weight and uterus index of rats are provided in Fig.2. Body weight (A) and uterus index (B) changes measured using an electronic scale. *P<0.01 compared with the sham group.

The uterus index was represented as the uterus weight divided by body weight. The data were expressed as mean ± SD (Fig. 2). As shown in Fig. 2, at week 0 of ovariectomy surgery, there was no significant difference in average weight among all the testing groups. However, the average weight of sham group was significantly lower than that of the other four study groups at week 4, at week 8, and at week 12. There was a general trend of longitudinal increase in the body weight for all the testing groups. The uterus index of the sham group was significantly higher than that of the other four study groups. However, there were no significant differences among the four study groups in both indices.

### Serum markers

The results for osteocalcin and CTX in the different groups at different time points are provided in Table 1. Compared with the Sham group osteocalcin (OC) rose in OVX+VEH group as well as in the other three treatment groups. The OVX+ALE group showed a significantly elevated level of OC at week 4 (+9%, p = 0.036), and the OVX+WBV group also exhibited a significantly elevated level of OC at week 8 (+13.4%, P<0.001) compared with the OVX+VEH group. However, OVX +WBV+ALE group showed no significant difference compared with the OVX+VEH group.

**Table 1:** Quantitative analysis of bone formation (osteocalcin) and resorption (CTX) markers of the serum levels in rats.

Groups			Weeks after surgery		
		0	4	8	12
**Osteocalcin (ng/mL)**	**SHAM**	30.13±1.04	30.52±1.35	28.78±1.04	28.10±1.69
		(3.45%)	(4.41%)	(3.61%)	(6.03%)
	**OVX+VEH**	——	36.92±1.54^a^	33.21±1.15^a^	35.29±2.38^a^
			(4.17%)	(3.45%)	(6.75%)
	**OVX+WBV**	——	35.49±1.00a	37.66±0.82^a+b^	34.45±1.40^a^
			(2.82%)	(2.19%)	(4.07%)
	**OVX +ALE**	——	39.87±0.82^a+b^	37.89±3.43^a^	37.48±2.12^a^
			(2.05%)	(9.05%)	(5.66%)
	**OVX+WBV+ALE**	——	35.61±1.27^a^	37.07±2.99^a^	36.85±2.78^a^
			(3.56%)	(8.06%)	(7.53%)
**CTX (ng/mL)**	**SHAM**	21.52±1.98	31.02±3.95	34.21±1.56	33.71±4.06
		(9.20%)	(12.74%)	(4.55%)	(12.04%)
	**OVX+VEH**	——	43.05±4.34^a^	44.09±3.14^a^	51.61±3.86^a^
			(10.07%)	(7.12%)	(7.47%)
	**OVX+WBV**	——	48.70±2.55^a^	42.15±4.13^a^	49.24±4.03^a^
			(5.24%)	(9.79%)	(8.18%)
	**OVX +ALE**	——	30.90±2.15^b^ (6.96%)	35.47±1.96^b^ (5.52%)	36.31±2.37^b^ (6.53%)
	**OVX+WBV+ALE**	——	30.56±2.86^b^	34.78±1.15^b^	38.45±2.27^b^
			(9.38%)	(3.32%)	(5.91%)

Values were the mean ± SD. The coefficient of variation of each bone biomarkers were presented under the data.

a : Significant difference from the SHAM group (p<0.05);

b : Significant difference from the OVX+VEH group (p<0.05).

With regard to bone resorption,CTX rose after ovariectomy. ALE treatment significantly prevented an increase in this serum marker. WBV alone treatment did not alter this effect. As shown in the table 1, the OVX+ALE and OVX+WBV+ALE group showed a significantly lower level of CTX compared to the OVX+VEH group at week 4 (−28.2%, P = 0.002; −29%, P = 0.001), week 8 (−19.6%, P = 0.002; −21.1%, P = 0.002) and week 12 (−29.6%, P<0.001; −25.5%, p<0.001). Moreover, at no point in the study was a significant difference found between the OVX+VEH and OVX+WBV groups for CTX.

### Micro-CT scanning

**Figure 3:**
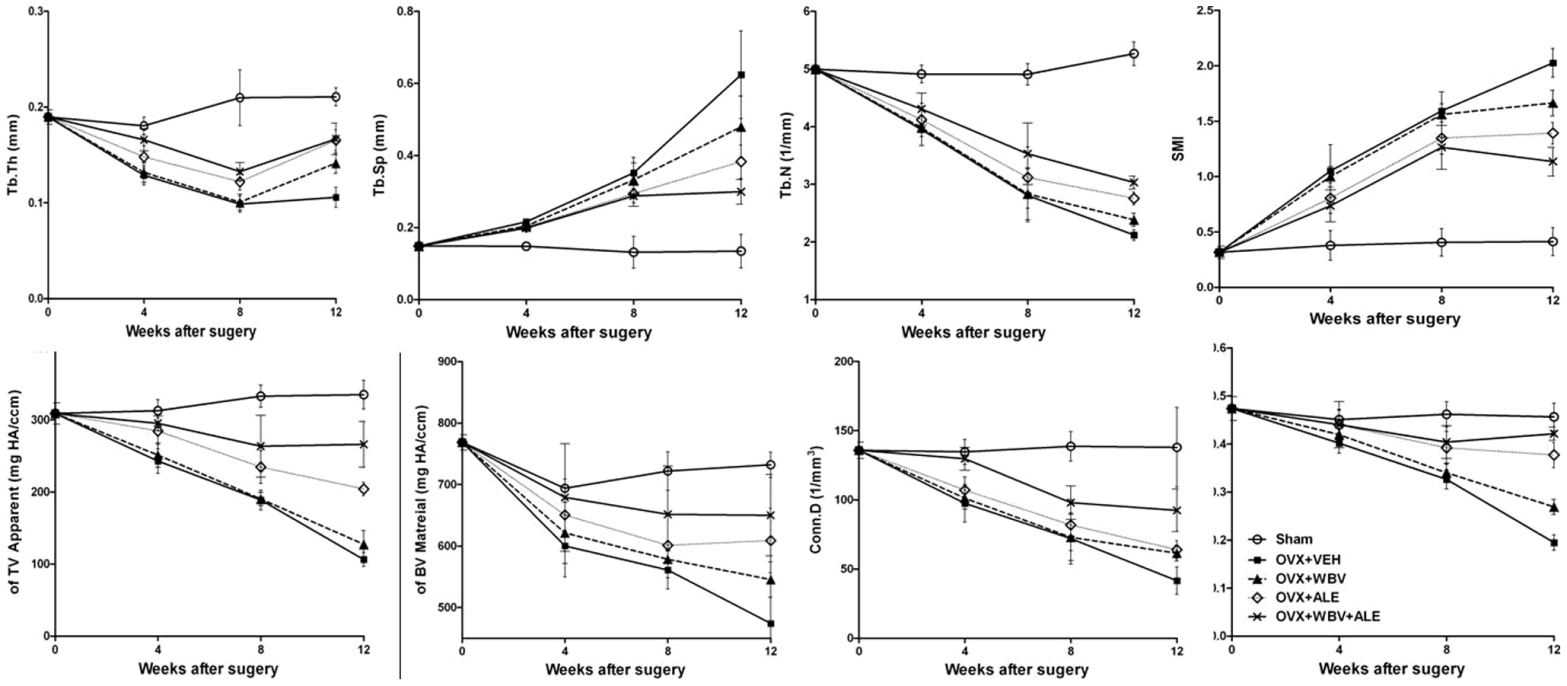
Morphological parameters of trabecular changes throughout the experiment as measured using μCT. Each value was derived from a single serial cross-section obtained from the left tibia of the rats. Data were presented as the mean ± SD.

**Figure 4:**
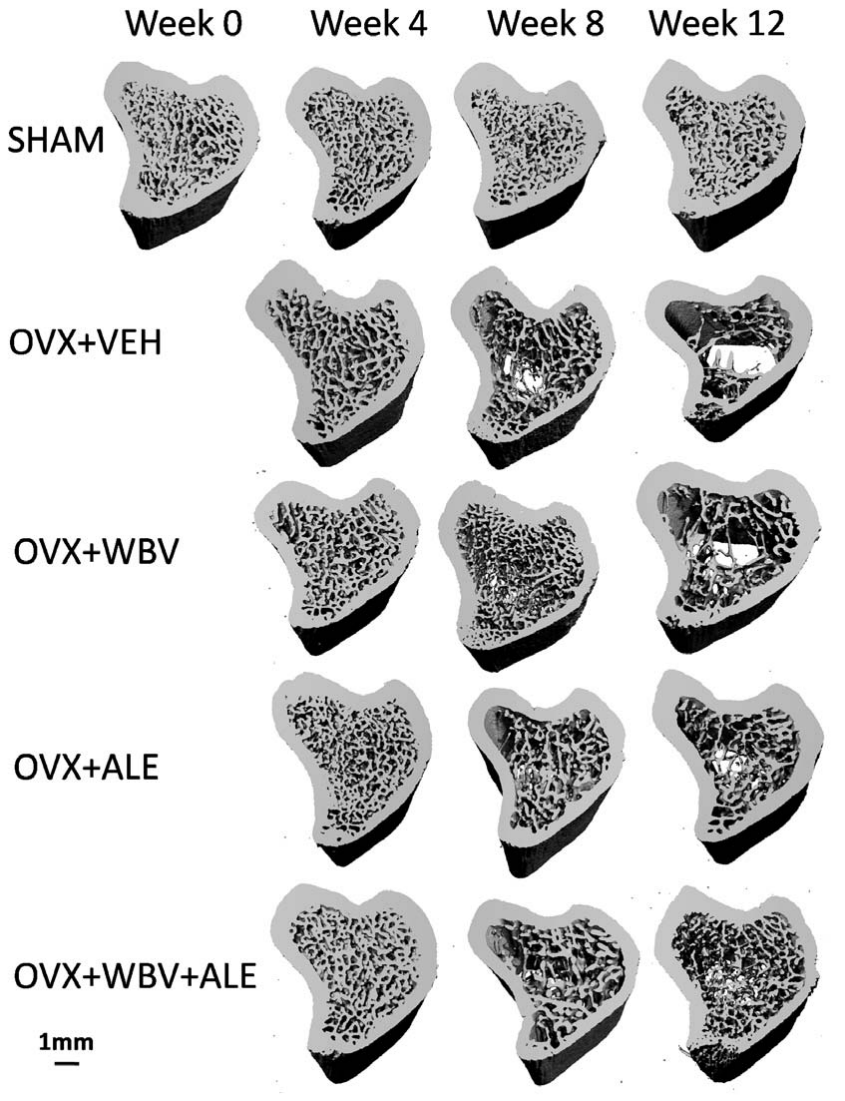
Three-dimensional μCT images of the metaphyseal tibia of the SHAM, OVX+VEH, OVX+WBV, OVX+ALE, and OVX+WBV+ALE-treated Sprague Dawley rats at the time of operation and follow-up measurements after four, eight and 12 weeks. Images were selected from animals with median cancellous BV/TV values.

The alteration of trabecular micro-architecture in the tibial metaphyseal region measured using μ CT is shown in Fig. 3, and the representative three-dimensional micro-CT reconstructions images are illustrated in Fig. 4.

At week 4, all of the morphological parameters [bone volume/tissue volume (BV/TV), structure model index (SMI), connective density (Conn. D), trabecular number (Tb. N), TV apparent, BV material, trabecular thickness (Tb. T h) and trabecular space (Tb. Sp)] showed no significant difference between the OVX+VEH and OVX+WBV groups, the OVX+VEH and OVX+ALE groups, the OVX+WBV and OVX+ALE groups, and the OVX+WBV and OVX+WBV+ALE groups (P>0.05). Furthermore, the OVX+ALE group elevated the BV/TV (+20%, p = 0.037) and Tb. T h (+23.6%, P = 0.006) compared with the OVX+VEH group at week 8.

Compared with the OVX+VEH group, nearly all tested indices were higher in the OVX+ALE group at week 12, such as the BV/TV (+73%, P<0.001), TV apparent (+89.9%, P<0.001), Tb. N (+29.9%, P<0.001), Tb. T h (+56.3%, P<0.001), and Conn. D (+53.6%, P  = 0.012), and the SMI (−31.3%, P<0.001) and Tb. Sp (−38.7%, P = 0.027) were lower in the OVX+ALE group. Moreover, compared with the OVX+VEH group, the BV/TV (+38.1%, P<0.001), Tb. N (+12.3%, P = 0.015), Tb. Th (+33.7%, P = 0.001), and Conn. D (+47.5%, P = 0.029) were higher, and the SMI (−17.9%, P = 0.003) was lower in the OVX+WBV group.

Compared with the OVX+WBV group, the BV/TV (+25.3%, p = 0.004), TV apparent (+60.3%, P<0.001), BV material (+28.4%, P = 0.044), Tb. N (+15.6%, P = 0.001), and Tb. T h (+16.9%, p = 0.037) were higher, and the SMI (−16.4%, p = 0.013) was lower in the OVX+ALE group at week 12. At week 12, significant differences were found for some tested indices between the OVX+ALE and OVX+WBV+ALE groups. Compared with the OVX+ ALE group, the BV/TV (+25%, p = 0.001), TV apparent (+30.4%, P = 0.032), Tb. N (+10%, P = 0.015), and Con. D (+44.5%, p = 0.042) were higher, and the SMI (−18.4%, p = 0.042) was lower in the OVX+WBV+ALE group.

### Biomechanical testing

**Figure 5:**
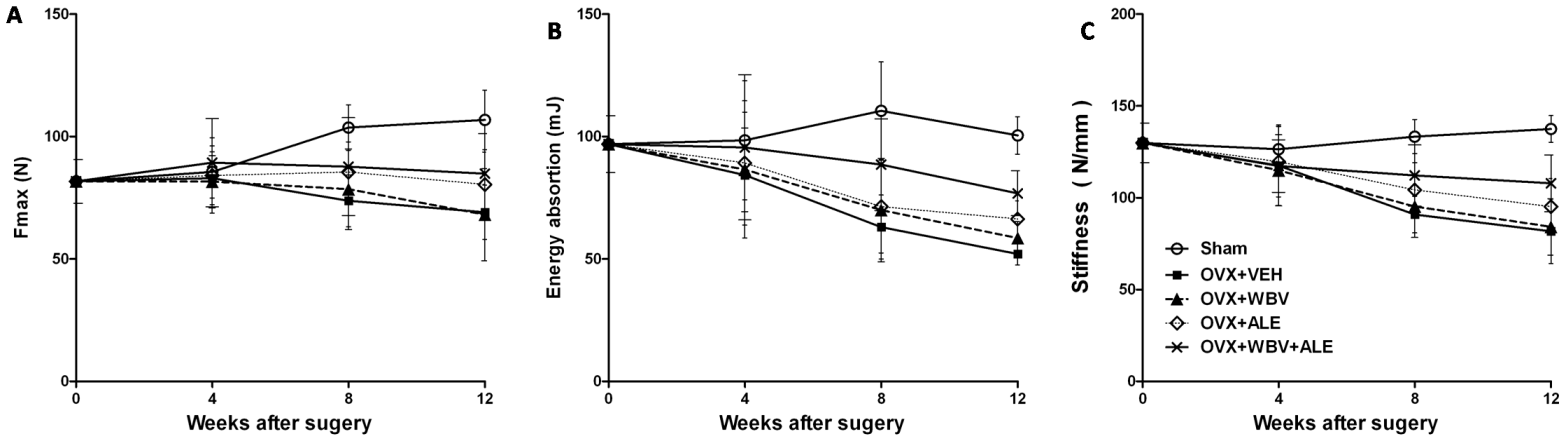
Change in the mechanical property at the metaphyseal tibia as measured using the three-point bending test. Each value was obtained from the right tibia of the rats. Data were presented as the mean ± SD.

The mean maximum load (F_max_), energy absorption and stiffness for all five groups at different time points are shown in Fig. 5. As the rats become older, the F_max_ of the sham group increased from 85.55±10.67 N at week 4 to 106.78±12.17 N at week 12 (+24.8%). Furthermore, the bone biomechanical parameters in the right tibiae were unchanged in all five groups at week 4. Moreover, the F_max_ in the right tibia showed no significant difference between the OVX+VEH and OVX+WBV groups (P = 1.000), the OVX+WBV and OVX+ALE groups (P = 1.000), and the OVX+ALE and OVX+WBV+ALE groups (P = 1.000) at week 8 or week 12. However, compared with the OVX+ VEH (69.80±9.23 N) group, the F_max_ were higher in the OVX+ALE group (89.07±4.96 N, +27.6%, P = 0.009) or in the OVX+WBV+ALE group (89.86±6.21 N, +28.7%, P = 0.008) at week 12.

As shown in Fig. 5, the bone energy absorption and bone stiffness in the tibia gradually decreased from four weeks to 12 weeks in the four study groups. At week 8, there were no significant differences for energy absorption in the four groups examined. At week 12, compared with the OVX+ VEH group (52.03±1.58 N), bone energy absorption were higher in both the OVX+ALE group (66.27±2.64 N, +27.4%, P = 0.040) and the OVX+WBV+ALE group (76.81±3.23 N, +47.6%, P = 0.003), but no significant differences were detected between the OVX+VEH and OVX+WBV groups (p = 0.815). As well, there were no significant differences for stiffness at any point in the four study groups examined.

## Discussion

This study investigated the effects of ALE, low-magnitude WBV and the combination of ALE and low-magnitude WBV on the ovariectomy-induced osteoporosis. In our study, the changes in bone quality were assessed through biomarkers, micro-CT, and three-point bending tests.

The SHAM group served as a negative control to illustrate normal bone growth over the course of the experiment. The OVX+VEH group was a positive control group to study changes occurring during the development of osteoporotic condition following the ovariectomy. Our results showed similar differences between the OVX+VEH and SHAM groups as have been previously characterized by micro-CT [21]–[25] and three point bending tests [26]. In our study, the OVX+VEH group had significantly higher levels of OC and CTX compared to the SHAM group. The uterus index of the sham group in our experiment was significantly higher than that of the other four groups. The effects of individual treatments such as ALE or low-magnitude WBV have also been extensively studied. However, to the best of our knowledge, there are no published studies investigating the effect of WBV combined with ALE on ovariectomized rats. Here we have shown the addition of low-magnitude WBV to ALE therapy improves trabecular architecture.

Bisphosphonates, such as ALE, have demonstrated compelling effects in the treatment of osteoporosis [27]. ALE has been shown to increase lumbar bone mineral density (BMD) and prevent vertebral fractures in post-menopausal females with osteoporosis [28]–[29]. Bisphosphonates are potent inhibitors of bone turnover [30]. These inhibitors act directly on osteoclasts to reduce bone resorption [31] and have been shown to reduce bone loss in established osteoporosis [32]–[33] and prevent bone loss in early menopause [34] or after surgical oophorectomy [35]. As shown in the table 1, vehicle-treated ALE significantly reduced the CTX serum level (−28.2%, at week 4; −19.6%, at week 8; −29.6%, at week 12) and increased the OC serum level (+9%, at week 4) compared with the OVX+VEH group. Osteocalcin, a non-collagenous protein synthesized by mature osteoblasts, is generally regarded as a specific marker of bone formation [36]–[37]. Furthermore, CTX, a collagen degradation product released by osteoclasts, is considered as a marker of bone resorption [38]–[39]. This study demonstrated that ALE inhibited osteoclast-mediated bone resorption and improved osteoblast-mediated bone formation.

Compared with histological analyses, micro-CT, can directly measure bone micro-architecture without relying on stereological models and is now regarded as the “gold standard” for the evaluation of bone morphology and micro-architecture in rats and other small animal models [40]. Micro-CT has provided evidence of a slight, local beneficial effect of ALE on trabecular bone. Our data fromμCT revealed that the micro-architectural bone parameters in the OVX+ALE group were significantly higher than that in the OVX+VEH group in nearly all tested indices at week 12. Thus, on the basis of these results ALE improve trabecular architecture.

The three-point bending test was utilized in this study to evaluate bone strength. The tibia samples were placed on a base that consisted of an aluminum block with one rounded edge-free notch on the top, and this design prevented slipping or tipping of the tibia with increasing punctual strength (Fig. 1B). At week 12, tibia treated with OVX+ALE had a significantly higher F_max_ and bone energy absorption compared to the OVX+VEH group. As such, ALE improved trabecular architecture and bone strength.

Therapeutic low-magnitude high-frequency whole-body vibration (WBV) has been shown to mitigate bone loss and improve neuromuscular function in specific studies [41]–[42]. It has been well-established that mechanical stimulation of bone formation is largely affected by the magnitude of the applied bone strain [43]–[47]. Many present studies show that WBV can subtly affect aging bone characteristics, depending on the region and vibrated parameters. For example, one study [48] showed that an acceleration of 0.5 g was favorable for bone density throughout most of the femur, whereas 1.5 g was largely ineffective in the femur, although it enhanced bone strength and stiffness in the radius. There was also a significant increase in the mineralizing surface in the distal femur for both vibration magnitudes. Another study showed [49] that WBV applied at increasing accelerations (0.1, 0.3, and 1.0 g) enhanced trabecular bone volume >30% in a non-dose-dependent fashion as assessed by histomorphometry in the proximal tibia of adult rats while no effect was observed at other bone sites such as vertebrae or femur. Thus the frequency and the amplitude of the sinusoidal vibration may have variable effects at different sites. The physical condition of the patient might also contribute to the efficacy of WBV [50]–[51]. Current literature [52]–[53] suggests a provisional consensus is building toward low-amplitude, high-frequency vibration as an effective and safe form of vibration. In contrast, high-amplitude vibration has unwanted consequences when chronically applied, as noted by a generation of studies correlating low back pain and other ailments to occupational vibration [54]. In animal models, bone formation rate has been shown to be retained in a model of disuse osteopenia when treated with 0.25 g WBV [55]. Similarly, in an oophorectomized rat model, 3.0 g WBV have a good effective on promoting the bone formation, as well as retention of muscle strength otherwise lost to the hormonal deficiency [42]. Garman et al. [56] reported that trabecular bone formation rate to bone surface ratio (BFR/BS) and mineralizing surface to bone surface ratio (MS/BS) were enhanced in female mice following 3 weeks of vibration. These enhancements in BFR/BS and MS/BS were acceleration-dependent with higher BFR/BS and MS/BS with the 0.3 g treatment vs. 0.6 g treatment.

In our study, we used the low-magnitude, low-amplitude high-frequency stimulus. WBV significantly increased the OC serum level (+13.4%, at week 8) compared with the OVX+VEH group. As shown in Fig. 3 and Fig. 4 the micro-architectural bone parameters (BV/TV, Tb. N, Tb. T h, Conn. D) in OVX+WBV group were significantly higher than that of the OVX+VEH group at week 12. This finding was similar to several published studies [57]–[58]. In conclusion, our results supported that WBV could improve bone formation and trabecular architecture.

To compare the different effect of the ALE drug and low-magnitude whole-body vibration on the bone parameters of ovariectomized rats was one aim of the study. Compared with the OVX+WBV group, nearly all of the tested indices (BV/TV, TV apparent, BV material, Tb. N, Tb. Th) were higher in the OVX+ALE group at week 12. Our result showed that the effect of alendronate on preventing bone loss and improving trabecular architecture was better than that of WBV.

Another aim of this study was to investigate the effect of WBV combined with ALE in ovariectomized rats. As shown in the table 1, WBV combined with ALE significantly reduced the CTX serum level (−29%, at week 4; −21.1%, at week 8; −25.5%, at week 12). In the OVX+ALE+WBV group, the trabecular BV/TV, Tb. N and Tb. T h were higher in the tibia compared to the OVX+VEH group (Fig. 3). At week 12, tibia treated with WBV+ALE had significantly higher F_max_ compared to the OVX+VEH group, and a significant difference was also found at week 12 for energy absorption between the OVX+WBV+ALE and OVX+VEH groups. As such, our study showed that WBV provide an additive effect to alendronate treatment which resulted in further reduction in bone mass and improvement in both trabecular architecture and bone strength.

The serum markers of bone turnover and bone biomechanical testing were not significant different between the OVX+ALE and OVX+WBV+ALE groups. However, in the OVX+ALE+WBV group, the trabecular BV/TV, TV apparent, Tb. N and Con. D were higher in the tibia compared to the OVX+ ALE group. This study demonstrated that WBV exercise enhanced the effect of ALE on the trabecular architecture in ovariectomized rats.

## Conclusion

Compared with WBV, ALE was more effective at preventing bone loss and to improve the trabecular architecture. However, WBV enhanced the effect of ALE in ovariectomized rats by inducing further improvements in trabecular architecture.
